# Carbapenem-Sparing Antimicrobial Stewardship in Internal Medicine: A Prospective Quasi-Experimental Before-and-After Pilot Implementation Study

**DOI:** 10.3390/antibiotics15090876

**Published:** 2026-09-07

**Authors:** Filippo Giorgio Di Girolamo, Filippo Mearelli, Donatella Denora, Ludovica Ilaria Carniel, Nicola Fiotti, Gianni Biolo, Dario Bianchini, Massimiliano Fabricci, Verena Zerbato, Stefano Di Bella, Chiara Roni

**Affiliations:** 1 Pharmacy Unit, Trieste University Hospital (ASUGI), 34125 Trieste, Italy; ludovica.carniel@asugi.sanita.fvg.it (L.I.C.);; 2 Department of Medical, Surgical and Health Science, University of Trieste, 34149 Trieste, Italyfiotti@units.it (N.F.);; 3 Unit of Internal Medicine, Clinica Medica, Trieste University Hospital (ASUGI), 34125 Trieste, Italy; filippome@libero.it; 4 Unit of Internal Medicine, Medicina Interna, Trieste University Hospital (ASUGI), 34125 Trieste, Italy; 5 Medical Directorate, Trieste University Hospital (ASUGI), 34128 Trieste, Italy; 6 Infectious Diseases Unit, Trieste University Hospital (ASUGI), 34125 Trieste, Italy

**Keywords:** antimicrobial stewardship, carbapenem-sparing strategy, time-limited dispensing, prospective reassessment, Internal Medicine, meropenem, fluoroquinolones, Defined Daily Dose, antimicrobial resistance

## Abstract

**Background/Objectives:** Carbapenem overuse contributes to antimicrobial resistance, but pragmatic stewardship models for high-complexity Internal Medicine wards remain underreported. We evaluated the feasibility and stewardship impact of a carbapenem-sparing intervention based on time-limited named-patient dispensing, pharmacist-supported reassessment, and mandatory infectious diseases reassessment for continuation beyond seven days, while assessing short-term clinical outcomes as exploratory safety signals. **Methods:** We conducted a prospective quasi-experimental before-and-after pilot implementation study in two Internal Medicine wards of a university hospital. Consecutive adult inpatients receiving a carbapenem or fluoroquinolone were enrolled during a 2-month control phase (standard care, *n* = 40) and a subsequent 2-month intervention phase (*n* = 38). Initial prescription remained at the discretion of the treating physician; targeted antibiotics were dispensed on a named-patient basis for a maximum initial duration of seven days, with pharmacist-supported reassessment on days 3 and 5 and mandatory infectious diseases reassessment for continuation beyond seven days. **Results:** Antibiotic therapy duration was shorter in the intervention group (median approximately 6 vs. 9 days, *p* = 0.0001), and antibiotic discontinuation by day 7 was more frequent than in controls (81.6% vs. 45.0%, *p* = 0.001). Total targeted antibiotic exposure decreased by approximately 40% (mean 5.9 vs. 10.0 Defined Daily Doses (DDD) per patient, *p* < 0.001), mainly driven by reduced meropenem use. No significant differences were observed in biomarker trajectories, clinical status at day 7, 30-day readmission or relapse, or 90-day mortality. **Conclusions:** A time-limited dispensing and reassessment model was feasible in Internal Medicine and was associated with lower carbapenem and targeted restricted-antibiotic exposure. No apparent short-term signal of clinical worsening was identified, although the study was not powered to establish safety, clinical non-inferiority, or equivalence. Larger multicentre studies using standardized stewardship metrics are needed.

## 1. Introduction

Antimicrobial resistance (AMR) has emerged as a critical global health threat. In 2019, almost 5 million deaths were associated with bacterial AMR, including 1.27 million directly attributable fatalities, surpassing the annual deaths from HIV/AIDS or malaria [1]. This burden makes AMR a leading cause of death worldwide, with the highest impacts in low-resource settings [2]. AMR also undermines modern medicine, rendering routine surgeries and treatments such as cancer chemotherapy much riskier [3]. Clinically, drug-resistant infections complicate treatment and increase patient mortality, particularly in hospital settings (e.g., intensive care units, ICUs) where multidrug-resistant (MDR) “ESKAPE” pathogens drive severe healthcare-associated infections [4]. Such difficult-to-treat infections lengthen hospital stays and necessitate expensive second-line therapies, escalating healthcare costs [2]. Economically, AMR’s impact is profound: global projections suggest a yearly gross domestic product (GDP) loss of up to US $3.4 trillion by 2030 without urgent action [5].

Antimicrobial stewardship (AMS) refers to coordinated interventions aimed at optimizing the use of antimicrobial agents to improve clinical outcomes while minimizing adverse effects, including the emergence of resistance [6]. Core AMS strategies in hospital settings include preauthorization of restricted antibiotics, prospective audit-and-feedback, guideline-based prescribing, and the optimization of dose, route and duration of therapy [7,8]. These interventions support rational antimicrobial use, particularly for agents of last resort.

Carbapenems and fluoroquinolones are broad-spectrum antibiotics indispensable for managing severe hospital-acquired infections caused by MDR organisms [9,10]. Carbapenems, β-lactam antibiotics, are essential for treating infections by both Gram-positive and Gram-negative pathogens, but their clinical effectiveness is increasingly compromised by resistance, primarily due to carbapenemases—β-lactamases capable of hydrolyzing and inactivating these antibiotics [9]. The progressive spread of carbapenem-resistant Enterobacterales and non-fermenters represents one of the most pressing threats in the hospital setting [11]. Fluoroquinolones, frequently prescribed for pneumonia, complicated urinary tract infections and bloodstream infections, face growing resistance driven by chromosomal mutations in bacterial enzymes such as DNA gyrase and topoisomerase IV, alongside plasmid-mediated quinolone resistance (PMQR) genes [12,13]. The misuse and overuse of these antibiotics—inappropriate empirical therapy, prolonged durations and inadequate dosing—further exacerbate resistance emergence [10,11]. Antimicrobial stewardship programs (ASPs) therefore strongly advocate for carbapenem- and fluoroquinolone-sparing strategies, emphasizing judicious prescribing and appropriate alternative regimens to preserve the efficacy of these critical antibiotics [10,14].

Recent studies have provided robust evidence supporting carbapenem-sparing strategies across various clinical settings, including ICUs, Internal Medicine wards and general hospitals. Key interventions evaluated include preauthorization policies, de-escalation protocols, comprehensive ASPs and alternative antibiotic regimens. Preauthorization—requiring stewardship team approval before carbapenem prescription—has consistently demonstrated significant reductions in carbapenem use. A 20-year study from a Japanese tertiary hospital showed sustained decreases in carbapenem consumption, correlated with reduced prevalence of resistant pathogens such as carbapenem-resistant *Pseudomonas aeruginosa* and *Acinetobacter baumannii*, without negatively impacting clinical outcomes or mortality [15]. Similarly, combined prospective audit-and-feedback with preauthorization in South Korea led to significant reductions in carbapenem use and stabilized resistance trends, notably for *Escherichia coli* and *Klebsiella pneumoniae* [16].

De-escalation strategies, switching from carbapenems to narrower-spectrum antibiotics once microbiological results or clinical improvement permitted, have effectively reduced treatment durations by approximately 2–5 days without compromising patient outcomes. A comprehensive narrative review indicated that de-escalation did not increase mortality or treatment failure, emphasizing its safety and effectiveness as an ASP component [17]. Hospital-wide stewardship programs further reinforce these findings: an ASP-led carbapenem-sparing initiative in Lebanon significantly improved prescribing appropriateness, increased pharmacist-led interventions and shifted usage toward narrower-spectrum agents [18]. Alternative antibiotic regimens, particularly newer β-lactam/β-lactamase inhibitor (BL/BLI) combinations, have also emerged as viable carbapenem-sparing options; a meta-analysis comparing new BL/BLI with carbapenems for bloodstream infections caused by extended-spectrum β-lactamase (ESBL)-producing organisms reported comparable clinical efficacy [19], and in high-risk neutropenic haematology patients carbapenem-sparing regimens were non-inferior in survival outcomes compared with carbapenem-based therapy [20]. Collectively, these findings indicate that carbapenem-sparing strategies can reduce antibiotic consumption and potentially lower resistance rates while preserving clinical outcomes.

In Italy, up to 40–50% of hospitalized patients in Internal Medicine receive at least one antibiotic [21], with carbapenems and fluoroquinolones accounting for nearly half of all inpatient antibiotic consumption [22]. This overuse is associated with an alarmingly high resistance burden, including approximately 30% of *K. pneumoniae* isolates resistant to carbapenems and over 40% of invasive *E. coli* resistant to fluoroquinolones [23], among the highest such rates in Europe [24,25,26,27]. While antimicrobial stewardship interventions in Italy have mostly targeted intensive care or Infectious Diseases units, general Internal Medicine wards remain underrepresented in prospective studies. Nonetheless, Internal Medicine departments play a pivotal role in the practical implementation of AMS policies, given the high rate of antibiotic prescriptions and the frail, elderly, multimorbid patients they care for, who are particularly vulnerable to infections and adverse drug events. Tailored AMS initiatives in this setting are therefore essential [28].

Against this background, we implemented a structured, multidisciplinary carbapenem-sparing stewardship strategy in a general Internal Medicine setting. The purpose was not to establish de novo that stewardship can reduce antibiotic use, an effect already supported by the literature, but to prospectively evaluate a pragmatic and reproducible workflow in high-complexity Internal Medicine wards. The model preserved the treating physician’s autonomy to initiate empirically reasoned carbapenem or fluoroquinolone therapy while combining time-limited named-patient dispensing, pharmacist-supported reassessment on days 3 and 5, and mandatory infectious diseases reassessment for continuation beyond seven days. This prospective quasi-experimental before-and-after pilot study compared standard care with this intervention, with the primary aim of reducing carbapenem (and, secondarily, fluoroquinolone) exposure while monitoring short-term clinical outcomes for any evident signal of worsening.

## 2. Results

### Baseline Characteristics

Baseline characteristics (Table 1) were well balanced between the control and intervention groups. No statistically significant differences were observed in demographics (age and sex distribution), prevalence of comorbid conditions, infection types, clinical severity at admission or initial antibiotic management strategies. Baseline inflammatory markers and liver enzymes did not differ between groups: CRP (C-reactive protein), procalcitonin, white blood cell (WBC) count, aspartate aminotransferase (AST) and alanine aminotransferase (ALT) levels were comparable (*p* > 0.05; see Supplementary Table S1). The prevalence of baseline renal impairment was also similar (chi-square test, *p* > 0.05). Recorded ESBL and *P. aeruginosa* risk-factor profiles were also comparable between groups (Table 1). Prior antibiotic exposure was similarly frequent in the two phases (65% of control vs. 71% of intervention patients, *p* = 0.76), with a comparable median duration of previous therapy (7 days in both groups), and, among patients who had received previous antibiotic therapy, empirical rather than microbiologically targeted treatment predominated in both groups (77% vs. 89%, *p* = 0.23). Class-specific prior antibiotic exposures, baseline resistance phenotypes and the detailed composition of combination regimens were not captured as structured variables and are therefore not reported.

**Figure 1:**
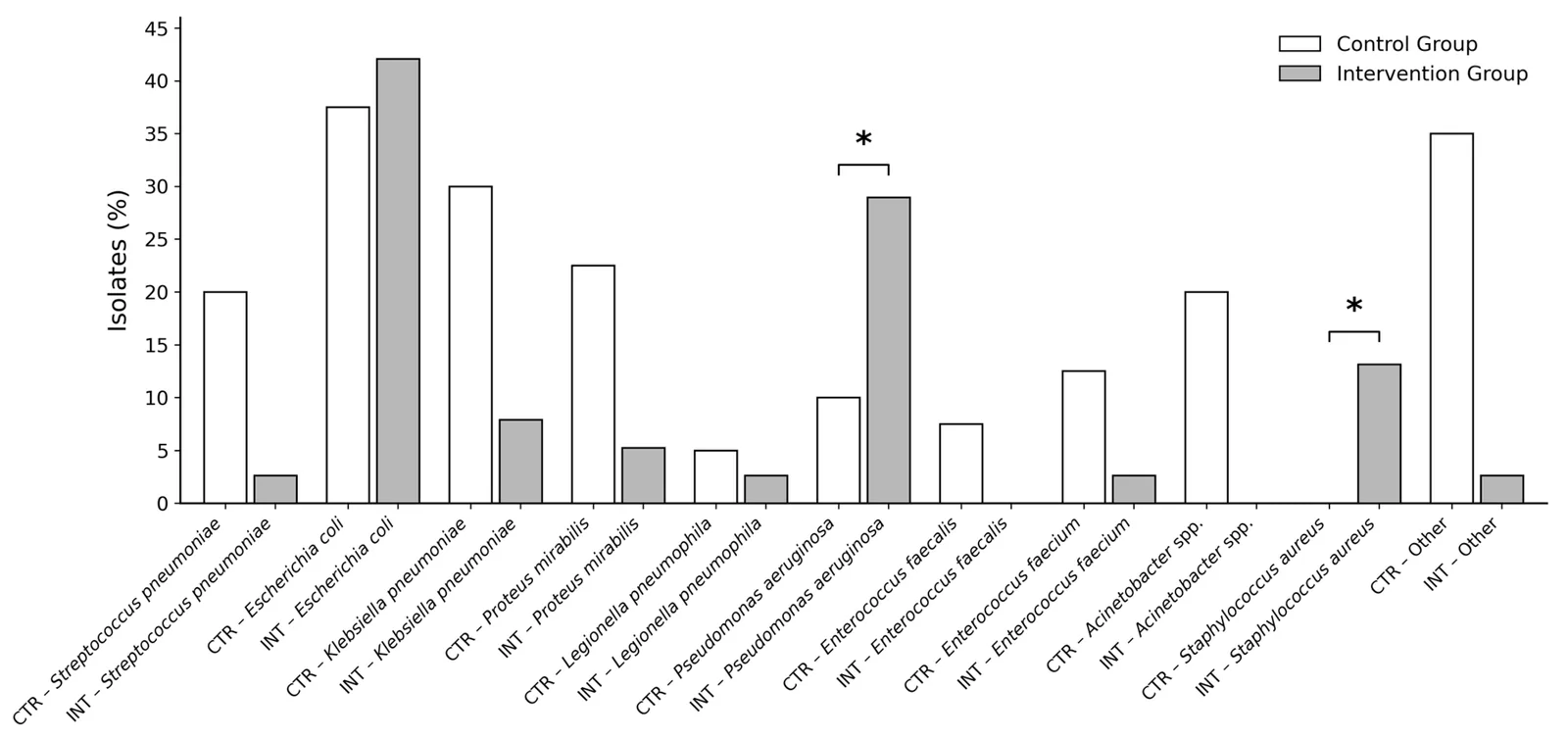
Overall pathogen distribution. Bar chart depicting the distribution of baseline microbiological isolates identified at hospital admission, comparing the control (CTR, white bars) and intervention (INT, grey bars) groups. Bar height indicates, for each group, the percentage of patients in whom the corresponding species was isolated; because some patients had more than one isolate, values within a group may sum to more than 100%. Organisms shown are *Streptococcus pneumoniae*, *Escherichia coli*, *Klebsiella pneumoniae*, *Proteus mirabilis*, *Legionella pneumophila*, *Pseudomonas aeruginosa*, *Enterococcus faecalis*, *Enterococcus faecium*, *Acinetobacter* spp., *Staphylococcus aureus* and other organisms. *P. aeruginosa* and *S. aureus* were more frequent in the INT group than in the CTR group, as indicated by the asterisks in the figure. * = *p* < 0.05 (Fisher’s exact test).

Baseline microbiological findings were descriptively compared between the control (*n* = 40) and intervention (*n* = 38) groups, and selected pathogen-specific differences were tested using Fisher’s exact test where appropriate (Figure 1).

As shown in Figure 1, *P. aeruginosa* and *Staphylococcus aureus* represented a higher proportion of baseline microbiological isolates in the intervention group than in controls. These baseline microbiological differences are considered further in the Discussion section.

**Figure 2:**
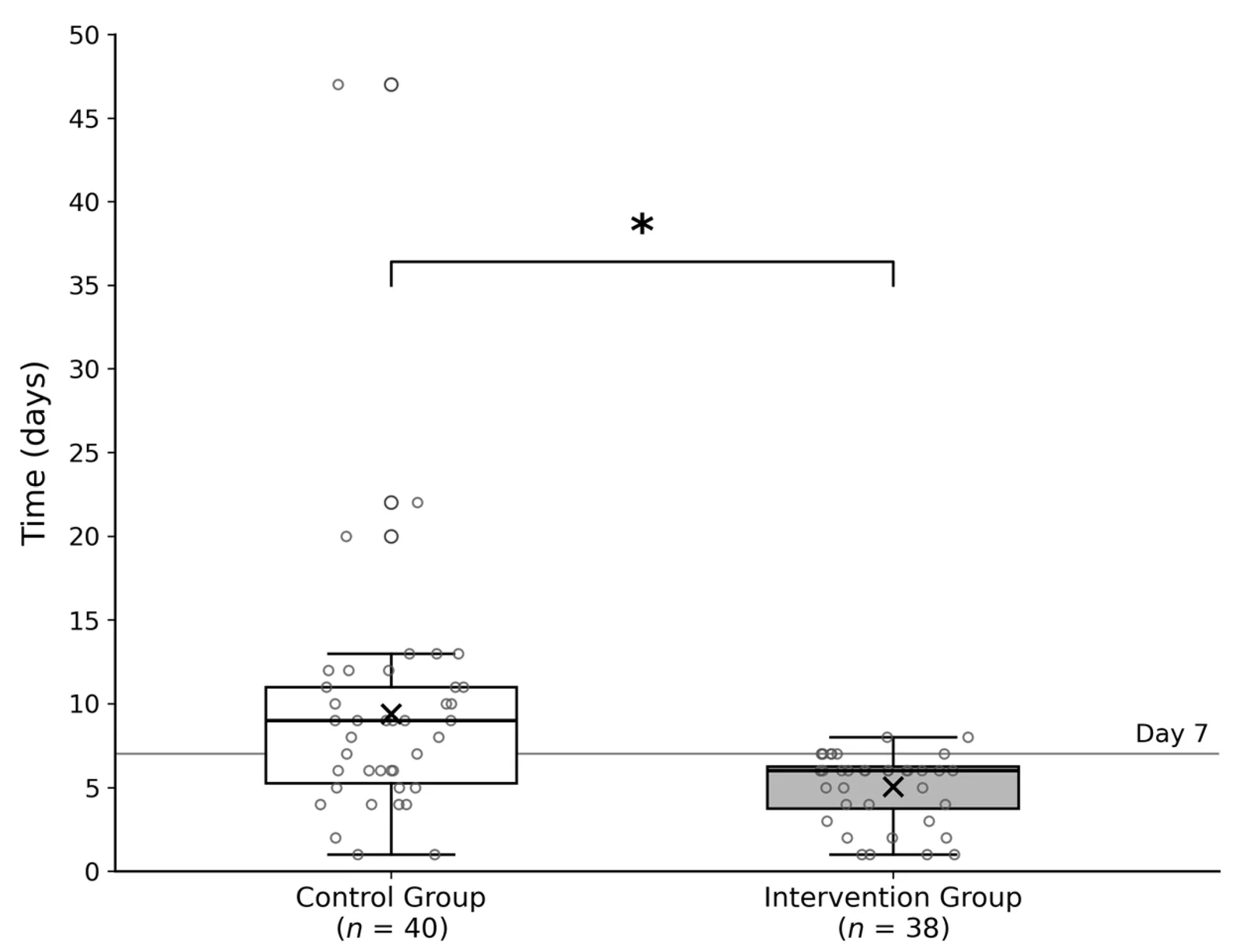
Duration of antibiotic therapy. Box-and-whisker plot comparing the number of days of antibiotic therapy from baseline (T0; initiation of the targeted antibiotic) to the final antibiotic dose in the control group (white box, *n* = 40) versus the intervention group (grey box, *n* = 38). Boxes indicate the interquartile range, the horizontal line within each box the median and the cross the mean; whiskers extend to the most extreme values lying within 1.5 × the interquartile range, and circles show individual patient values. The horizontal reference line marks day 7, the maximum treatment duration covered by a single named-patient dispensation. * = *p* < 0.05.

**Figure 3:**
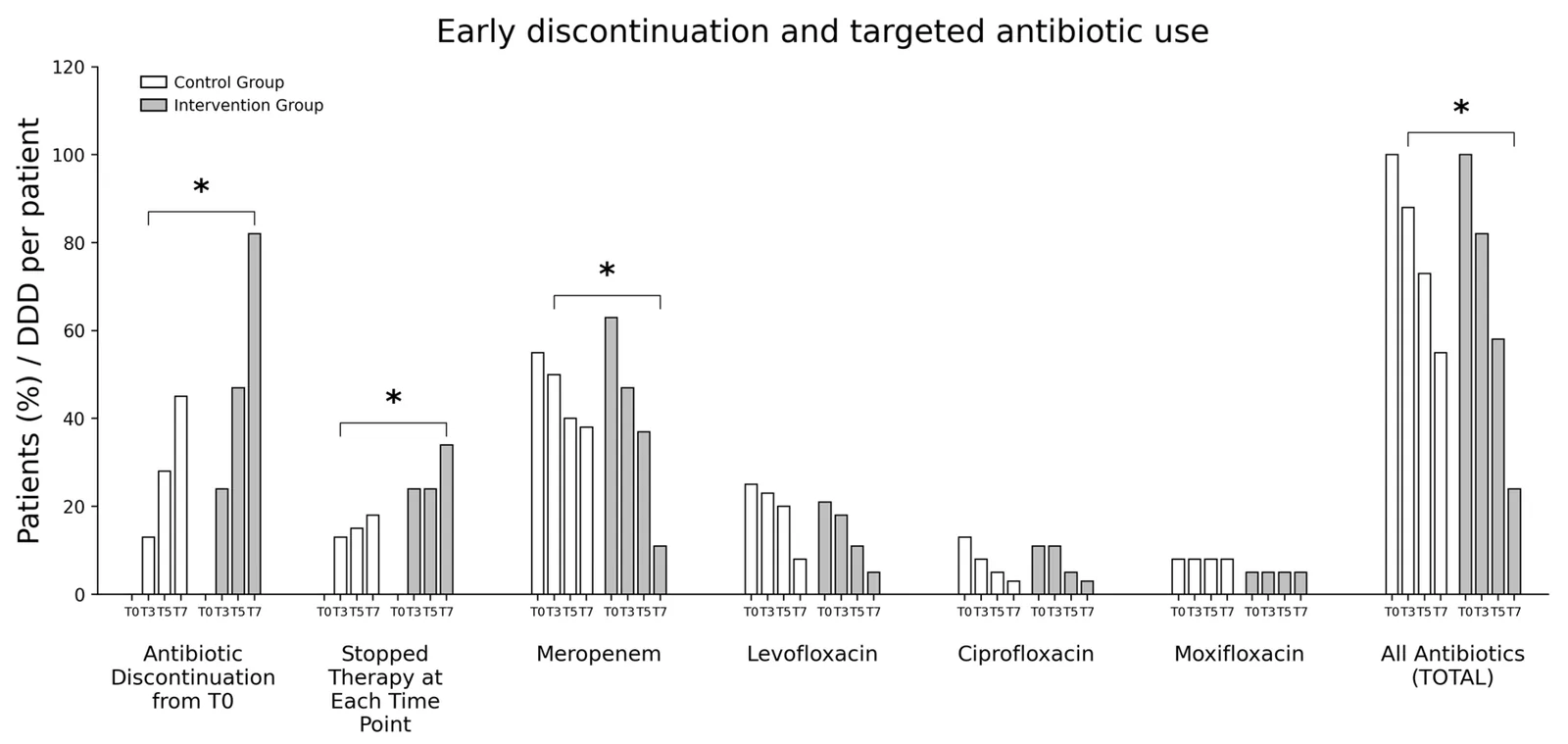
Early antibiotic discontinuation and targeted antibiotic use in the control versus intervention groups. Within each category, bars show values at therapy initiation (T0) and after 3 (T3), 5 (T5) and 7 (T7) days. The first two categories show the cumulative percentage of patients who had discontinued antibiotic therapy since T0 and the percentage who stopped therapy at each individual time point. The remaining categories show the percentage of patients still receiving meropenem, levofloxacin, ciprofloxacin, moxifloxacin and any targeted antibiotic (All Antibiotics, TOTAL) at each time point. White bars represent the control group and grey bars the intervention group. * = *p* < 0.05.

The total duration of antibiotic therapy from T0 to the final antibiotic dose was significantly shorter in the intervention group (Figure 2). The median duration of antibiotic therapy was approximately 6 days (interquartile range, IQR ≈ 4–6) in the intervention group, compared with approximately 9 days (IQR ≈ 5–11) in the control group (*p* = 0.0001). As shown in Figure 3, by day 3 after initiation of the targeted antibiotic, 23.7% of patients in the intervention group had discontinued antibiotic therapy, compared with 12.5% in the control group (*p* = 0.24). At day 5, the proportion of patients off antibiotics had increased to 47.4% in the intervention group versus 27.5% in controls (*p* = 0.10). By day 7, 81.6% of intervention patients had stopped antibiotics, significantly more than the 45.0% of control patients (*p* = 0.001). Thus, the intervention phase was associated with earlier antibiotic discontinuation, reaching statistical significance by one week.

Overall antibiotic consumption, measured as Defined Daily Doses per patient, was substantially lower in the intervention arm. On average, intervention patients received about 5.9 DDD of antibiotics during the hospital stay, compared with approximately 10.0 (9.97) DDD in control patients—roughly a 40% reduction (*p* < 0.001). Meropenem use was particularly reduced: among meropenem-treated patients, meropenem consumption averaged approximately 11 DDD per treated patient in the control group versus approximately 5 DDD in the intervention group (this denominator is restricted to meropenem-treated patients and therefore differs from the all-patient DDD reported above). This pattern was consistent with a reduction concentrated in carbapenem exposure. No significant differences in fluoroquinolone consumption were observed between groups—levofloxacin and ciprofloxacin DDD per patient were similar in the two cohorts—and moxifloxacin use, while lower in the intervention group, involved few patients and did not reach significance.

No patient received imipenem–cilastatin or norfloxacin during the study periods; these prespecified eligible agents are therefore not displayed in Figure 3.

A separate request-level operational registry was used to describe implementation processes during the November–December 2024 intervention period in the two participating wards (Supplementary Table S3). The registry contained 38 request records; 37/38 (97.4%) resulted in dispensing, whereas one request (2.6%) was not dispensed because treatment had already been discontinued. Six request records (15.8%) contained at least one subsequent registry entry, comprising 10 follow-up events: nine additional dispensing events and one documented treatment discontinuation without further dispensing. These request-level operational records were analysed separately from the patient-level clinical cohort. Consistently with a workflow in which authorization applied to continuation rather than to initiation, the single non-dispensing event followed a continuation request submitted on day 9 of therapy.

**Figure 4:**
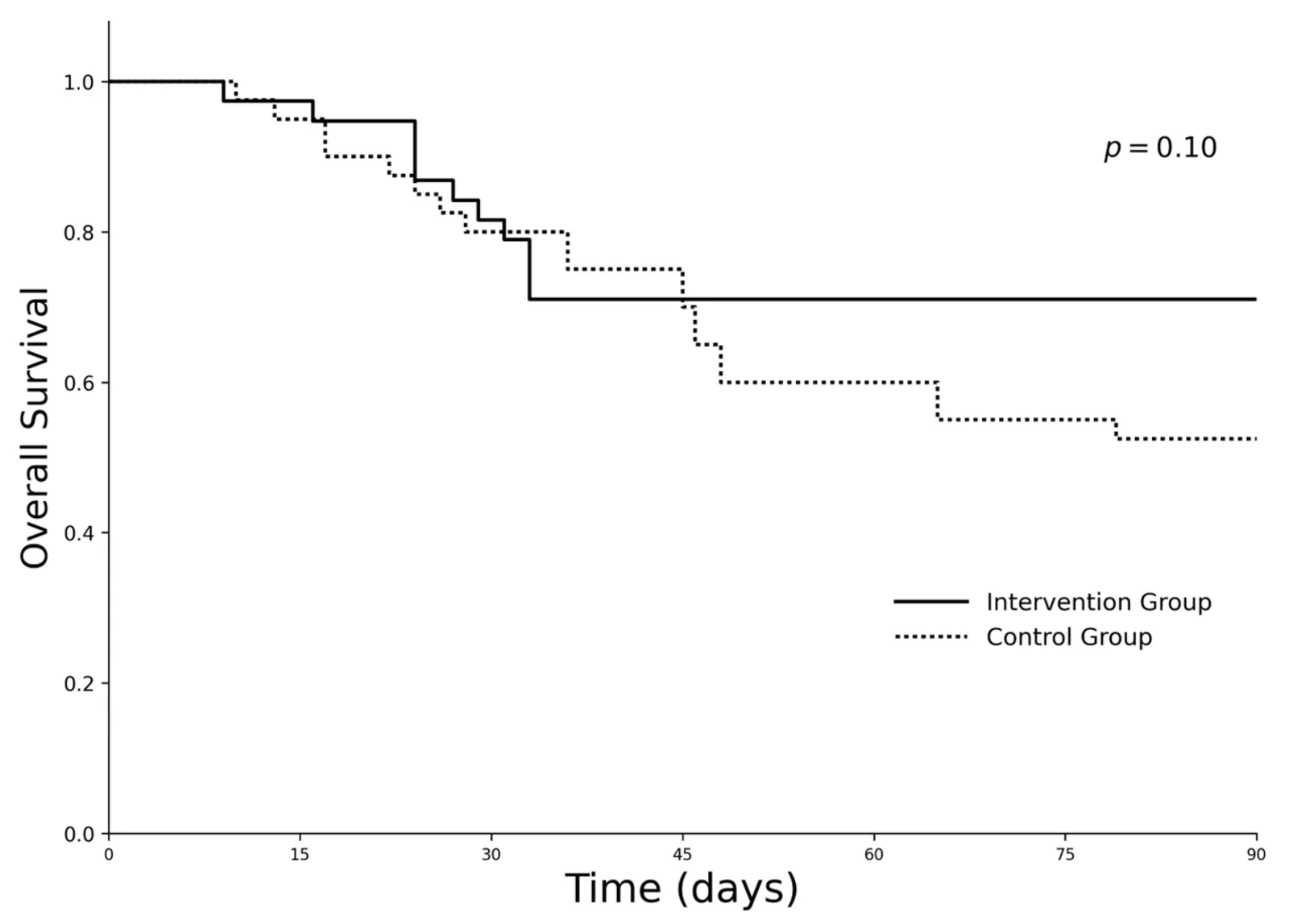
Ninety-day overall survival from hospital admission. Control group: dashed line; intervention group: solid line. The endpoint is 90-day all-cause mortality from the day of admission, with patients alive at 90 days censored at day 90. Survival curves were estimated using the Kaplan–Meier method, and the two groups were compared using a log-rank test. Ninety-day survival did not differ significantly between groups (*p* = 0.10).

Kaplan–Meier analysis of 90-day survival (Figure 4), as well as clinical status at day 7 and 30-day outcomes (Table 2), indicated no significant difference between the control and intervention groups.

Overall, no significant differences over time were observed between the control and intervention groups for most parameters. Body temperature and inflammatory markers (including CRP and procalcitonin) declined similarly in both groups (Supplementary Table S1; *p* > 0.05). Platelet counts increased over time in both groups without significant group differences. Differential counts of neutrophils, lymphocytes, monocytes, eosinophils and basophils showed no significant interaction between group and time (all *p* > 0.05). Notably, the total WBC count decreased more markedly in the intervention group than in controls by day 5, a difference that reached statistical significance (*p* = 0.03). Renal function (serum creatinine) and liver enzymes (AST, ALT, alkaline phosphatase, bilirubin) evolved similarly in both groups, with no significant group–time interactions (all *p* > 0.05).

## 3. Discussion

In this prospective quasi-experimental before-and-after pilot implementation study, the implementation of a pharmacist- and infectious diseases (ID) physician-led carbapenem-sparing antimicrobial stewardship protocol in an Internal Medicine setting was feasible and was associated with a substantial reduction in targeted restricted-antibiotic exposure. The study should be interpreted as a pragmatic hospital stewardship quality-improvement implementation project rather than as a formal clinical efficacy trial.

Patients were enrolled consecutively rather than through formal randomization; nonetheless, the two groups were clinically comparable at baseline with respect to critical characteristics such as age, sex and essential clinical parameters [29,30]. At baseline, however, a higher proportion of *Pseudomonas aeruginosa* and *Staphylococcus aureus* isolates was observed in the intervention group. Such microbiological imbalances can arise by chance in non-randomized designs and do not inherently indicate systematic bias; methodological guidance cautions against over-interpreting baseline differences that lack clear prognostic significance [30]. All patients received guideline-driven empirical antibiotic therapy, and clinical outcomes—including length of hospital stay, biomarker trajectories, readmissions and relapse rates—did not differ significantly between groups despite these initial microbiological differences. We did not perform pathogen-stratified analyses, so the extent to which baseline microbiology influenced antibiotic-use metrics could not be quantified. This imbalance may reflect random variability in a small non-randomized cohort; nonetheless, because these pathogens influence antibiotic choice and expected treatment duration, residual microbiological confounding cannot be excluded and is considered further in the section Strengths and Limitations.

Total antibiotic duration was significantly shorter in the intervention arm, with the median therapy length reduced (intervention ≈ 6 days vs. control ≈ 9 days). The reduction in targeted restricted-antibiotic exposure was driven chiefly by lower carbapenem consumption—reflected in both the total Defined Daily Doses per patient and the meropenem dose per treated patient—rather than by a lower proportion of patients started on a carbapenem, which was in fact numerically higher in the intervention group (63% vs. 55%, *p* = 0.46). The reduction in exposure therefore reflects shorter and more frequently reassessed courses rather than fewer carbapenem initiations. Total fluoroquinolone exposure per included patient was somewhat lower in the intervention group, although per-treated-patient levofloxacin and ciprofloxacin doses were similar and the difference did not reach statistical significance. The modest difference in fluoroquinolone use partly reflects the intentional, judicious use of these agents for oral step-down therapy. Fluoroquinolones (e.g., ciprofloxacin and levofloxacin) are currently among the few oral antibiotics active against *P. aeruginosa*, making them valuable for transitioning stable patients to outpatient treatment [31]. Appropriate use of oral fluoroquinolones in susceptible *P. aeruginosa* infections enables earlier hospital discharge and de-escalation from intravenous therapy, reducing inpatient days and catheter-associated risks while still effectively treating the infection [32]. Preserving a defined role for fluoroquinolones in such targeted scenarios supports stewardship goals, even if the overall reduction in fluoroquinolone use is modest, and indicates that these agents are being reserved for critical step-down needs rather than overused.

The muted difference in fluoroquinolone use between groups can also be explained by a historical decline in fluoroquinolone prescribing in Italy and across Europe. The European Medicines Agency has restricted the use of fluoroquinolones because of disabling and potentially permanent adverse effects, recommending that they be avoided in mild-to-moderate infections when alternatives are available [33]; national regulators and Italian guidelines have similarly discouraged broad fluoroquinolone use in favour of narrower-spectrum drugs [34]. Reflecting this trend, Italian stewardship reports have documented marked drops in fluoroquinolone consumption [35]: one hospital ASP reported a 22.9% reduction in fluoroquinolone DDDs after guideline dissemination [34], and another study reported fluoroquinolone use falling by approximately 48% between 2019 and 2021 [22]. When comparing fluoroquinolone consumption (DDD per 100 bed-days) in the same two-month period one year earlier (September–October 2023) with the control phase of our study (September–October 2024) in the same Internal Medicine wards, we observed a reduction from approximately 9.3 to 5.8 DDD per 100 bed-days. In other words, fluoroquinolone use was already declining, which likely dampened any additional impact of our intervention on this class. In contrast, carbapenem use has not declined nationally: point-prevalence surveys and utilization data consistently show persistently high use of broad-spectrum “last-line” antibiotics in Italy, with meropenem ranking among the top five inpatient antibiotics [22], particularly in high-acuity wards such as Internal Medicine. The impact of our program was therefore most pronounced for carbapenem use, consistent with the absence of an ongoing national downward trend for this class [22,34].

Consistent with this interpretation, exploratory post-implementation pharmacy surveillance data from January to September 2025, compared with the same period in 2024, showed lower aggregate carbapenem consumption expressed as DDD per 100 bed-days (total carbapenems, 4.57 to 3.93; meropenem, 3.61 to 3.17). This was accompanied by increased use of selected carbapenem-sparing alternatives, including piperacillin-tazobactam (15.27 to 16.87), fosfomycin (2.59 to 2.99), ceftolozane–tazobactam (0.73 to 1.64) and temocillin (0 to 0.15). These ecological data support a shift away from carbapenem exposure but should be interpreted cautiously because they are aggregate consumption indicators and not patient-level outcome data.

Within hospital stewardship, preauthorization and prospective audit-and-feedback (PAF) are both regarded as core strategies but differ in timing and function. Preauthorization acts before prescription or dispensing, whereas PAF acts after prescription and is generally more educational and less restrictive of prescriber autonomy [6]. The intervention evaluated here did not use classical front-end preauthorization for treatment initiation. Instead, the treating physician retained responsibility for the initial empirically reasoned prescription, while named-patient dispensing was time-limited to a maximum initial duration of seven days. Pharmacist-supported reassessment occurred on days 3 and 5, and continuation beyond seven days required mandatory reassessment by an infectious diseases specialist. The model therefore combined prospective review with a defined stewardship checkpoint for prolonged therapy.

The time-limited dispensing and reassessment framework was associated with lower consumption of targeted antibiotics compared with the pre-intervention control group. The smaller antibiotic exposure of intervention patients was not accompanied by an apparent worsening of the available short-term clinical outcomes. Key infection biomarkers (CRP, procalcitonin, WBC count and neutrophils) evolved similarly in both groups (Supplementary Table S1), and there were no statistically significant differences in clinical status at day 7, 30-day infection relapse, rehospitalization, or 90-day mortality. These findings are exploratory safety signals only and should not be interpreted as evidence of clinical safety, equivalence, or non-inferiority.

The carbapenem-sparing strategy evaluated here should be interpreted as selective de-escalation and careful patient selection rather than indiscriminate replacement of carbapenems. In clinically stable patients with a controlled source of infection and an available antibiogram, alternatives such as trimethoprim–sulfamethoxazole (TMP/SMX) or, when appropriate and with documented susceptibility, ciprofloxacin or levofloxacin may represent valid options for targeted or oral step-down therapy, particularly in urinary tract infections caused by ESBL-producing Enterobacterales (ESBL-E) [36]. Piperacillin-tazobactam may be considered only in selected low-inoculum scenarios—chiefly some cases of pyelonephritis or complicated urinary tract infection and, with further caution, biliary-source infections with adequate source control and clinical stability—whereas carbapenems remain the preferred choice in severe extra-urinary infections, uncontrolled bloodstream infection or septic shock [36,37]. For cephamycins, including cefoxitin, current clinical evidence remains limited and does not support routine use; any such use should be regarded as selective and provisional, dependent on local susceptibility data and close stewardship supervision [36]. Accordingly, our intervention is best understood as a model of patient selection, de-escalation and shortening of therapy duration rather than a policy of automatic carbapenem substitution.

Our findings align with a growing body of literature showing that ASPs can markedly decrease antibiotic usage without clear worsening of reported clinical outcomes. A recent global meta-analysis (52 studies, 2010–2020) found that ASP implementation was associated with a 28% reduction in overall antibiotic consumption, with the largest reductions in broad-spectrum classes: fluoroquinolone use decreased by approximately 42% and carbapenem use by approximately 31% on average [38]. The pattern observed in our intervention, particularly the reduction in carbapenem exposure, is consistent with these estimates.

Targeted restriction through preauthorization appears particularly effective. A 2025 study in Poland implemented a hospital-wide preauthorization policy for fluoroquinolones and observed an almost eight-fold reduction in fluoroquinolone use on a urology ward (from 358.9 to 43.4 DDD per 1000 patient-days), together with a 25% absolute decrease in fluoroquinolone-resistant infections [39]. Multiple studies have reported that pharmacist-driven stewardship interventions achieve such reductions without evident adverse effects on clinical outcomes. Xia et al. [40] evaluated a pharmacist-led multifaceted ASP in a tertiary hospital and found significant reductions in antibiotic consumption with no increase in mortality or length of stay. A systematic review by Dighriri et al. [41] further reinforces the role of pharmacists in ASPs, reporting that pharmacist-led interventions improved appropriate prescribing and reduced unnecessary antibiotic use and costs without increasing readmission rates. In our study, the absence of any rise in 30-day readmissions or 90-day mortality is consistent with, but does not prove, the possibility that judicious antibiotic curtailment can be implemented without an apparent short-term signal of clinical worsening [42]. Our data describe how such a workflow can be operated in a high-complexity general ward and provide the process and consumption metrics required to design an adequately powered multicentre evaluation.

### Strengths and Limitations

Strengths of this study include its prospective, real-world design, targeting a prevalent problem—excessive carbapenem and targeted restricted-antibiotic use—in Internal Medicine, a setting representative of routine hospital care. The stewardship protocol was multifaceted while preserving prescriber autonomy at treatment initiation: targeted agents were dispensed on a named-patient basis for a time-limited initial course, treatment was reassessed on days 3 and 5 with pharmacist support, and prolonged courses required infectious diseases specialist reassessment. This workflow leveraged complementary professional expertise and was designed for routine implementation in high-complexity general wards.

This study was not designed as a formal non-inferiority trial. The modest sample size, the absence of randomization, the quasi-experimental before-and-after nature of the comparison, and the lack of a prespecified non-inferiority margin preclude any formal conclusion of clinical non-inferiority [43]. The clinical findings should therefore be interpreted as exploratory pilot evidence—a reduction in antibiotic exposure, particularly to carbapenems, with no apparent short-term signal of clinical worsening within the available follow-up—rather than as proof of safety or equivalence. Antibiotic use was reported primarily as DDD per patient; future studies should adopt standardized denominators such as DDD per 100 patient-days and days of therapy (DOT) per 1000 days present, together with prespecified stewardship endpoints, adequate statistical power, and prospectively structured process metrics.

Further limitations should be acknowledged. The study was conducted at a single centre with a relatively small cohort, which may limit generalizability. The two-phase design lacked a concurrent control group, leaving open the possibility of unmeasured confounding and secular or seasonal trends, although no other antimicrobial stewardship initiatives, new formulary restrictions, infection-control policies, diagnostic pathways, or major organizational changes affecting antibiotic prescribing were introduced in the participating wards during the study period. The higher proportions of *P. aeruginosa* and *S. aureus* in the intervention group may have influenced antibiotic selection and treatment duration; residual microbiological confounding therefore cannot be excluded. Clinical and laboratory characteristics were assessed at all scheduled time points. The T7 improved/stable/worsened category was prospectively entered in the case report form by the treating physician; no validated scale or independent adjudication procedure was used for this pragmatic assessment. Outcomes were assessed only in the short term (up to 30 days for readmission and relapse, and 90 days for mortality), so longer-term effects on AMR or late adverse events could not be evaluated. The operational registry permitted reporting of request-level dispensing and follow-up events, but infectious diseases consultation status, recommendation adherence, authorization turnaround time, and formal de-escalation were not captured as structured variables and could not be quantified reliably. Finally, the local resistance ecology and organizational context may differ elsewhere, limiting broad extrapolation.

## 4. Materials and Methods

### 4.1. Study Design and Setting

We conducted a prospective quasi-experimental, two-phase before-and-after pilot implementation study in two Internal Medicine wards of Cattinara Hospital (Trieste, Italy). The study was undertaken as a hospital antimicrobial stewardship quality-improvement/pilot implementation initiative aimed at optimizing the use of restricted antibiotics in routine care. The study comprised a control phase (September–October 2024) and an intervention phase (November–December 2024), allowing comparison of outcomes before versus after introducing the stewardship intervention. Each ward was a 41-bed Internal Medicine unit at a university-affiliated hospital. No other antimicrobial stewardship initiatives, new formulary restrictions, infection-control policies, diagnostic pathways, or major organizational changes affecting antibiotic prescribing were introduced in the participating wards during the study period. This pilot study was undertaken as part of a hospital antimicrobial stewardship program aimed at optimizing antibiotic use in accordance with national guidelines and best practice [24,44].

### 4.2. Participants

The study population included 78 consecutive adult inpatients admitted to two Internal Medicine wards during the study period who received antibiotic therapy with a carbapenem or a fluoroquinolone. Eligible antibiotics comprised carbapenems (meropenem or imipenem–cilastatin) and fluoroquinolones (ciprofloxacin, levofloxacin, moxifloxacin or norfloxacin). Patients were enrolled consecutively as they initiated one of the targeted antibiotics, with no other exclusion criteria. This yielded 40 patients in the control phase and 38 patients in the intervention phase. Each patient was followed from the start of antibiotic therapy through discharge, with follow-up at 30 days post-discharge.

### 4.3. Intervention Approach

During the intervention phase, the decision to initiate empirical or empirically reasoned treatment with a targeted carbapenem or fluoroquinolone remained under the responsibility of the treating physician and did not require prior stewardship authorization. Targeted antibiotics were no longer available as unrestricted ward stock and were dispensed on a named-patient basis, with the initial supply limited to a maximum treatment duration of seven days. Treatment appropriateness and clinical evolution were reassessed on days 3 and 5 with support from the on-ward clinical pharmacist, taking into account the available clinical and microbiological information, dose appropriateness, and opportunities for discontinuation or de-escalation. If the treating physician considered continuation beyond seven days necessary, further dispensing required mandatory reassessment by an infectious diseases specialist. Thus, the intervention was designed primarily to promote early reassessment and prevent unnecessarily prolonged exposure rather than to restrict the initial empirical prescription.

In the control phase, antibiotic prescribing followed usual care: carbapenems and fluoroquinolones could be obtained from ward stock without time-limited named-patient dispensing, scheduled pharmacist-supported reassessment, or mandatory infectious diseases reassessment for continuation beyond seven days.

### 4.4. Outcomes and Data Collection

The primary outcomes were stewardship (antibiotic-use) indicators. The principal patient-level stewardship outcome was total exposure to the targeted restricted antibiotics, expressed as DDD per included patient. Secondary stewardship indicators included duration of antibiotic therapy and the timing of antibiotic discontinuation, expressed as the proportion of patients off antibiotics by days 3, 5 and 7 after initiation of the targeted antibiotic. Antibiotic consumption was quantified using the Defined Daily Dose (DDD) [45]. The DDD-to-number-of-patients ratio is an indicator frequently used in pharmacoepidemiological databases to express treatment intensity and total exposure over a defined study period [45]; when the actual dose used equals the DDD, the DDD per patient also approximates the number of treatment days. Aggregate post-implementation surveillance data, when discussed, were summarized separately as DDD per 100 bed-days and were not treated as patient-level outcome data. The duration of therapy for each patient’s antibiotic course (in days) was recorded to assess whether the intervention achieved shorter, appropriate treatment durations.

Exploratory short-term clinical outcomes were assessed as safety signals rather than as a formal test of non-inferiority. These comprised: (1) the evolution of infection biomarkers during treatment—C-reactive protein (CRP), procalcitonin, white blood cell (WBC) count and neutrophils—recorded at therapy initiation (T0) and after 3 (T3), 5 (T5) and 7 (T7) days, and every 7 days thereafter until discharge; and (2) clinical outcomes, including clinical status at T7 relative to T0, rehospitalization and infection relapse within 30 days, and all-cause mortality assessed up to 90 days. Clinical and laboratory characteristics were evaluated at all scheduled time points. At T7, the treating physician prospectively recorded clinical status in the case report form as improved, stable, or worsened relative to T0. This pragmatic three-level assessment was not based on a validated clinical scale and was not independently adjudicated. Relapse was defined as recurrence or persistence of the index infection requiring further medical evaluation or antibiotic treatment within 30 days. All laboratory data were available within one day of admission.

All patient-level data were collected from medical records, laboratory systems, and pharmacy dispensing logs. In addition, the stewardship pharmacist maintained a separate operational registry of named-patient requests and subsequent dispensing events during the intervention phase. This registry was used to describe request-level implementation indicators and was analysed separately from the patient-level clinical cohort. Because initial targeted-antibiotic prescription did not require stewardship approval, the registry was not interpreted as a conventional approved/rejected preauthorization log. Infectious diseases consultation status and adherence to recommendations were not recorded as structured variables. Follow-up at 30 days post-discharge was conducted by reviewing readmission records and outpatient notes, or by telephone contact when necessary, to determine clinical outcomes. Ninety-day all-cause mortality was ascertained from hospital records and available administrative follow-up data, with patients alive at 90 days censored at day 90 for survival analysis.

### 4.5. Ethics and Governance

The stewardship intervention was implemented as an institutional ASUGI antimicrobial stewardship procedure. Ethics and consent details are reported in the Institutional Review Board Statement and Informed Consent Statement.

### 4.6. Statistical Analysis

Continuous variables (e.g., age, biomarker levels, antibiotic days and DDDs per patient) were summarized as medians with interquartile ranges (IQR), and categorical variables (e.g., 30-day outcome events) as counts and percentages. For between-group comparisons, non-parametric and chi-square tests were used as appropriate. Continuous outcomes not normally distributed were compared between the control and intervention groups using the Mann–Whitney U test, while categorical outcomes were compared using the chi-square or Fisher’s exact test. To analyse longitudinal changes in biomarkers over the course of therapy, a repeated-measures General Linear Model (GLM) was used to assess the interaction between time and group. All hypothesis tests were two-tailed, and a *p*-value < 0.05 was considered statistically significant. No formal sample-size calculation or non-inferiority margin was prespecified; given the pilot nature of the study, clinical outcomes were analysed descriptively as exploratory safety signals. Statistical analyses were performed using IBM SPSS Statistics, version 21.0 (IBM Corp., Armonk, NY, USA), and all results were reviewed by the study team for clinical as well as statistical significance.

## 5. Conclusions

In a real-world Internal Medicine setting, a multidisciplinary carbapenem-sparing stewardship strategy based on time-limited named-patient dispensing, pharmacist-supported reassessment, and mandatory infectious diseases reassessment for continuation beyond seven days was feasible and was associated with a marked reduction in carbapenem and targeted restricted-antibiotic exposure. No apparent signal of clinical worsening emerged within the available short-term follow-up, although the study was not powered to establish clinical safety, non-inferiority, or equivalence. The principal contribution of this work is the pragmatic, reproducible description of a stewardship model transferable to high-complexity general wards. These findings should be confirmed in multicentre studies with prespecified stewardship outcomes, standardized consumption metrics, prospectively structured process indicators, and adequate statistical power.

## Figures and Tables

**Table 1:** Patient characteristics on admission.

	Control (*n* = 40)	Intervention (*n* = 38)	*p*
Age (years)	80 (76–82.8)	81 (73.5–87.5)	0.44
Sex (male/female, %)	52/48	61/39	0.50
Charlson Comorbidity Index (CCI)	6 (5–8)	6.5 (5–8)	0.81
FIB-4 index	2.0 (1.45–3.19)	1.6 (1.08–2.64)	0.11
Hepatic Risk Category (H/M/L, %)	43/43/15	24/32/45	0.07
Baseline SOFA score	3 (1–4)	2 (1–3.25)	0.25
NEWS score	4.5 (4–7)	3.5 (2–6)	0.10
**Infection site**			
Lung (%)	50	48	0.10
Blood (%)	10	5	
Urinary tract (%)	13	35	
Skin (%)	10	0	
Ear (%)	8	5	
Biliary tract (%)	5	0	
Other (%)	0	5	
Not defined (%)	5	3	
MRSA risk factors (yes/no, %)	18/83	8/92	0.31
ESBL risk factors (yes/no, %)	38/62	37/63	1.00
*P. aeruginosa* risk factors (yes/no, %)	58/43	63/37	0.65
Carbapenem as first-line therapy (%)	55	63	0.46
Previous antibiotic therapy (%)	65	71	0.76
Prior empirical therapy (% of previously treated patients)	77	89	0.23

Control group *n* = 40; intervention group *n* = 38. CCI = Charlson Comorbidity Index; FIB-4 = Fibrosis-4 index; H/M/L = high/medium/low hepatic risk category (based on FIB-4 cut-offs); MRSA = methicillin-resistant *Staphylococcus aureus*; ESBL = extended-spectrum β-lactamase; SOFA = Sequential Organ Failure Assessment; NEWS = National Early Warning Score. Continuous variables are reported as median (interquartile range); categorical variables as percentages. Significance was defined as *p* < 0.05. Clinical risk criteria for MRSA, ESBL and *P. aeruginosa* are reported in Supplementary Table S2.

**Table 2:** Clinical status at day 7 and clinical outcomes during follow-up.

Outcome	Control (*n* = 40)	Intervention (*n* = 38)	*p*
Improved (%)	48	47	0.69
Worsened (%)	23	16	
Stable (%)	30	37	
Length of hospital stay (days)	18.5 (10–36.75)	17 (9.75–26.75)	0.55
Home therapy duration (days)	3 (2–9)	3 (1.25–9.25)	0.53
Readmission within 30 days (no/yes)	32/7	33/3	0.31
Relapse within 30 days (no/yes)	36/3	33/3	1.00

Continuous variables (length of stay and therapy durations) are expressed as median (interquartile range) and were compared using the Mann–Whitney U test. Categorical variables (including 30-day readmission and relapse) were compared using Fisher’s exact test. “Improved”, “Worsened” and “Stable” denote patient clinical status at T7 relative to T0, prospectively entered in the case report form by the treating physician. “No/Yes” values for readmission and relapse indicate the number of patients without versus with the event within 30 days. Readmission and relapse data were unavailable for 1 control patient and 2 intervention patients; these comparisons were performed on available cases. For multi-category variables, *p* values refer to the overall comparison across categories.

## Data Availability

The data presented in this study are available on request from the corresponding author due to privacy, legal and institutional restrictions. The data are not publicly available because they contain institutional clinical data subject to applicable data protection regulations.

## Supplementary Materials

The following supporting information can be downloaded at: https://www.mdpi.com/article/10.3390/antibiotics15090876/s1, Table S1: Evolution of clinical and laboratory parameters over time; Table S2: Clinical risk criteria for MRSA, ESBL-producing Enterobacterales and *Pseudomonas aeruginosa*; Table S3: Request-level implementation indicators during the intervention phase [46].

## Abbreviations

ALT	Alanine Aminotransferase
AMR	Antimicrobial Resistance
AMS	Antimicrobial Stewardship
ASP	Antimicrobial Stewardship Program
AST	Aspartate Aminotransferase
BL/BLI	β-Lactam/β-Lactamase Inhibitor
CCI	Charlson Comorbidity Index
CRP	C-Reactive Protein
DDD	Defined Daily Dose
DOT	Days of Therapy
ESBL	Extended-Spectrum β-Lactamase
ESBL-E	ESBL-producing Enterobacterales
FIB-4	Fibrosis-4 (liver fibrosis index)
ICU	Intensive Care Unit
ID	Infectious Diseases
IQR	Interquartile Range
MDR	Multidrug-Resistant
MRSA	Methicillin-Resistant Staphylococcus aureus
NEWS	National Early Warning Score
PAF	Prospective Audit and Feedback
PMQR	Plasmid-Mediated Quinolone Resistance
SOFA	Sequential Organ Failure Assessment
TMP/SMX	Trimethoprim–Sulfamethoxazole
WBC	White Blood Cell (Count)
