# Supplementary material for: Carbapenem-Sparing Antimicrobial Stewardship in Internal Medicine: A Prospective Quasi-Experimental Before-and-After Pilot Implementation Study
Source: Antibiotics (Basel). 2026 Sep 7;15(9):876. doi: 10.3390/antibiotics15090876 (PMC13603156; doi:10.3390/antibiotics15090876)
Supplement: Supplementary file 1 [file antibiotics-15-00876-s001.zip › antibiotics-4442476-supplementary.pdf]

SUPPLEMENTARY MATERIAL

Supplementary Table S1. Evolution of clinical and laboratory parameters over time

Parameter	Group	Time				p
		T0	T3	T5	T7	
**Temperature (°C)**	**Control**	36.5 (36.4–36.7)	36.5 (36.4–36.5)	36.5 (36.4–36.5)	36.5 (36.4–36.5)	**0.92**
	**Intervention**	36.5 (36.5–36.7)	36.5 (36.4–36.5)	36.5 (36.4–36.5)	36.5 (36.5–36.5)	
**C-reactive protein (mg/L)**	**Control**	123.2 (44.4–201.9)	79.2 (44.8–123.6)	57.8 (28.4–79.7)	68.4 (28.6–86.6)	**0.55**
	**Intervention**	114.3 (77.7–162.0)	70.8 (32.9–124.2)	67.7 (20.6–90.0)	91.2 (22.9–159.7)	
**Procalcitonin (μg/L)**	**Control**	0.50 (0.19–1.02)	0.30 (0.17–1.06)	0.17 (0.08–0.25)	0.18 (0.09–0.53)	**0.61**
	**Intervention**	0.17 (0.11–1.57)	0.35 (0.11–4.95)	0.24 (0.11–0.99)	0.17 (0.09–0.48)	
**Platelet count (×10³/μL)**	**Control**	232 (176–340)	239 (197–356)	246 (221–348)	295 (199–359)	**0.47**
	**Intervention**	244 (174–351)	259 (155–313)	303 (221–393)	263 (218–343)	
**WBC count (×10³/μL)**	**Control**	9.44 (7.79–15.53)	9.45 (6.39–13.22)	9.84 (6.15–11.36)	10.12 (6.00–13.17)	**0.03**
	**Intervention**	9.32 (7.33–13.01)	7.84 (5.95–8.70)	7.46 (6.14–8.87)	7.70 (5.55–10.93)	
**Neutrophils (×10³/μL)**	**Control**	6.38 (4.61–10.79)	7.82 (5.67–9.59)	6.72 (4.13–8.53)	7.46 (4.67–10.69)	**0.68**
	**Intervention**	6.69 (5.55–9.21)	4.58 (4.29–5.77)	5.12 (3.05–6.37)	8.60 (8.60–9.64)	
**Lymphocytes (×10³/μL)**	**Control**	1.31 (0.91–1.88)	0.97 (0.80–1.66)	1.32 (0.64–2.05)	1.28 (1.07–1.58)	**0.08**
	**Intervention**	0.81 (0.58–1.79)	1.47 (0.67–2.36)	1.32 (0.91–2.36)	3.00 (1.31–3.00)	
**Monocytes (×10³/μL)**	**Control**	0.70 (0.38–0.96)	0.74 (0.55–0.98)	0.74 (0.40–0.89)	0.71 (0.51–0.90)	**0.74**
	**Intervention**	0.76 (0.52–0.93)	0.58 (0.35–0.83)	0.58 (0.42–0.77)	1.26 (0.37–1.26)	
**Eosinophils (×10³/μL)**	**Control**	0.15 (0.06–0.24)	0.07 (0.00–0.31)	0.12 (0.03–0.39)	0.05 (0.01–0.33)	**0.13**
	**Intervention**	0.02 (0.01–0.10)	0.15 (0.06–0.44)	0.11 (0.06–0.30)	0.38 (0.07–0.38)	
**Basophils (×10³/μL)**	**Control**	0.05 (0.03–0.06)	0.03 (0.01–0.05)	0.05 (0.02–0.05)	0.05 (0.03–0.06)	**0.20**
	**Intervention**	0.03 (0.02–0.04)	0.03 (0.02–0.05)	0.04 (0.02–0.05)	0.08 (0.03–0.08)	
**Creatinine (mg/dL)**	**Control**	1.18 (0.85–1.50)	1.02 (0.83–1.50)	1.08 (0.79–1.49)	1.08 (0.81–1.39)	**0.33**
	**Intervention**	0.89 (0.65–1.44)	0.79 (0.62–1.18)	0.73 (0.60–0.94)	0.95 (0.74–1.29)	
**AST (U/L)**	**Control**	35.0 (21.8–60.3)	32.0 (20.5–91.5)	N/R	25.5 (19.8–152.8)	**0.89**
	**Intervention**	18.0 (14.5–44.5)	22.5 (14.3–46.0)	18.5 (17.0–36.3)	N/R	
**ALT (U/L)**	**Control**	35.5 (17.5–49.8)	25.0 (18.0–65.0)	N/R	20.0 (6.5–51.3)	**0.76**
	**Intervention**	17.0 (9.5–37.5)	20.5 (7.3–35.0)	14.5 (7.3–30.8)	N/R	
**Alkaline phosphatase (U/L)**	**Control**	90 (85–167)	149 (120–365)	N/R	238 (83–391)	**0.44**
	**Intervention**	101 (64–141)	80 (55–170)	N/R	N/R	
**Total bilirubin (mg/dL)**	**Control**	0.96 (0.73–1.42)	1.22 (0.53–2.19)	N/R	0.89 (0.54–6.41)	**0.52**
	**Intervention**	0.47 (0.36–0.68)	0.55 (0.31–1.12)	0.50 (0.27–0.75)	N/R	

Footnote. Values are median (interquartile range). T0 = therapy initiation; T3, T5, T7 = days 3, 5 and 7 of therapy. N/R = not recorded (the test was not performed at that time point). WBC = white blood cell; AST = aspartate aminotransferase; ALT = alanine aminotransferase. p-values are from a repeated-measures general linear model evaluating differences in the trajectory over time between the control and intervention groups. Statistical significance was defined as $p < 0.05$.

Supplementary Table S2. Clinical risk criteria for MRSA, ESBL-producing Enterobacterales and Pseudomonas aeruginosa[46]

**Pathogen**	**Risk factor**
MRSA (methicillin-resistant *Staphylococcus aureus*)	Previous MRSA colonization and/or infection (within the past 12 months)
	Haemodialysis or peritoneal dialysis
	Presence of a central venous catheter or other long-term intravascular catheter
	Multiple antibiotic courses (e.g., ≥5–10 days in the last 30 days), especially fluoroquinolones or cephalosporins
ESBL-producing Enterobacterales	Previous ESBL colonization and/or infection (within the past 12 months)
	Prolonged hospitalization (median ≈10 days), particularly in ICU, long-term care facilities, hospices or high-endemicity wards
	Multiple antibiotic courses (≥5 days in the last 30 days), especially fluoroquinolones or cephalosporins
	Indwelling urinary catheter
	PEG feeding tube
Pseudomonas aeruginosa	Previous *P. aeruginosa* colonization and/or infection (within the past 12 months)
	Multiple antibiotic courses (≥5 days in the last 30 days), especially fluoroquinolones
	Structural lung disease with recurrent infections (e.g., bronchiectasis)
	Cystic fibrosis
	Prolonged systemic corticosteroid therapy (>6 weeks)
	Poorly controlled diabetes / acute decompensation and/or diabetic foot
	Indwelling urinary catheter
	Advanced age (>80 years)
	Immunosuppression

Supplementary Table S3. Request-level implementation indicators during the intervention phase

**Process indicator**	**%**
Requests resulting in dispensing	97.4
Requests not dispensed because treatment had already been discontinued	2.6
Request records with ≥1 subsequent registry entry	15.8
**Among subsequent registry events:**	
Additional dispensing	90.0
Discontinuation without further dispensing	10.0

Footnote. Process indicators were derived from the operational request registry of Clinica Medica and Medicina Interna 9 during the intervention period. Percentages in the first three rows are calculated on all 38 request records. Six request records (15.8%) generated at least one subsequent registry entry, accounting for 10 subsequent events overall. Percentages in the two indented rows are calculated on these 10 subsequent events: 90.0% resulted in additional dispensing and 10.0% documented treatment discontinuation without further dispensing. The operational registry was used exclusively to describe implementation processes and was not used to define the analytic study cohort.
